# Hepatic Ischemia and Reperfusion Injury and Trauma: Current Concepts

**DOI:** 10.5812/atr.12501

**Published:** 2013-08-01

**Authors:** Dimitrios Papadopoulos, Thomas Siempis, Eleni Theodorakou, Georgios Tsoulfas

**Affiliations:** 1 1st Department of Surgery, Aristotle University of Thessaloniki, Papageorgiou General Hospital, Thessaloniki, Greece

**Keywords:** Reperfusion Injury, Ischemia, Pathophysiology, Prevention

## Abstract

**Context:**

Ischemia-reperfusion injury is a fascinating topic which has drawn a lot of interest in the last several years. Hepatic ischemia reperfusion injury may occur in a variety of clinical situations. These include transplantation, liver resection, trauma, and vascular surgery.

**Evidence Acquisition:**

The purpose of this review was to outline the molecular mechanisms underlying hepatic I/R injury and present the latest approaches, both surgical and pharmacological, regarding the prevention of it. A comprehensive electronic literature search in MEDLINE/PubMed was performed to identify relative articles published within the last 2 years.

**Results:**

The basic mechanism of hepatic ischemia – reperfusion injury is one of blood deprivation during ischemia, followed by the return of flow during reperfusion. It involves a complex series of events, such as mitochondrial deenergization, adenosine-5'-triphosphate depletion, alterations of electrolyte homeostasis, as well as Kupffer cell activation, oxidative stress changes and upregulation of proinflammatory cytokine signaling. The great number of variable pathways, with several mediators interacting with each other, leads to a high number of candidates for potential therapeutic intervention. As far as surgical approaches are concerned, the modification of existing clamping techniques and the ischemic preconditioning are the most promising techniques till recently. In the search for novel techniques of protecting against hepatic ischemia reperfusion injury, many different strategies have been used in experimental models. The biggest part of this research lies around antioxidant therapy, but other potential solutions have been explored as well.

**Conclusions:**

The management of hepatic trauma, in spite of the fact that it has become increasingly nonoperative, there still remains the possibility of hepatic resection in the hepatic trauma setting, especially in severe injuries. Hence, clinicians should be familiar with the concept of hepatic ischemia-reperfusion injury and respond appropriately and timely.

## 1. Context

Ischemia reperfusion injury (IRI) is defined as the phenomenon during which cellular damage in an organ, caused by hypoxia, is paradoxically exacerbated after the restoration of oxygen delivery (1). It is a dynamic process which involves the two interrelated phases of local ischemic insult and inflammation-mediated reperfusion injury (2). This concept occurs in several organ systems such as the central nervous system, liver, heart, lung, intestine, skeletal muscle, and kidney (3). If severe enough, the inflammatory response after IRI may even result in the systemic inflammatory response syndrome (SIRS) or the multiple organ dysfunction syndrome (MODS) (4). Hepatic IRI is a frequent and major complication in clinical practice, which compromises liver function and increases postoperative morbidity, mortality, recovery, and overall outcome. Liver, being an organ with high energy requirements, is highly dependent on oxygen supply and susceptible to hypoxic or anoxic conditions (5). Hepatic IRI can be categorized into warm and cold ischemia. Warm ischemia occurs in the setting of transplantation, trauma, shock, and elective liver surgery, in which hepatic blood supply is temporarily interrupted. It may also occur in some types of toxic liver injury, sinusoidal obstruction and Budd-Chiari syndrome (6). Cold storage ischemia occurs during organ preservation before transplantation. Numerous factors contribute to hepatic IRI, including Kupffer cells (KC) activation, oxidative stress and upregulation of proinflammatory cytokine signaling (7). This variety of mechanisms, contribute to various extents to the overall pathophysiology. Hence, it is difficult to achieve effective protection by targeting individual mediators or mechanisms and plentiful strategies reducing IRI, both clinical and experimental, have been extensively studied (8). Unfortunately, the outcomes of promising experimental studies cannot be always applied in the clinical setting. As far as the importance of hepatic IRI in trauma is concerned, despite the fact that the management of hepatic trauma has become increasingly nonoperative, there still remains the possibility of hepatic resection in the hepatic trauma setting, especially in severe injuries (9). Intraoperative cessation of hepatic blood supply, by a variety of clamping maneuvers, is sometimes necessary during resection and inevitably exposes the liver to warm IRI. Furthermore, the liver with its role as the biochemical factory of sorts for the organism, as well as its anatomic and physiologic position, is vulnerable to the ischemia which is frequently encountered in patients with trauma. In this paper we would review the latest knowledge regarding the pathophysiology of hepatic ischemia reperfusion injury and present current and future options, both surgical and pharmacological, to attenuate it.

## 2. Evidence Acquisition

A review of the most recent literature was conducted using PubMed asa search engine with the focus on papers dealing with ischemia/reperfusion of the liver, especially relating to hepatic trauma or similar situations. Additionally, emphasis was given on identifying the most recent data on the mechanisms involved in hepatic ischemia/reperfusion injury.

## 3. Results

### 3.1. Current Knowledge of the Pathophysiology of Hepatic I/R Injury

During an ischemic period, several functional changes occur at the cellular level that promote cell injury (10). More specifically, a decrease in oxidative phosphorylation, results in Adenosine-5'-triphosphate (ATP) depletion and derangements in calcium homeostasis (11). The lack of oxygen to hepatocytes during ischemia also causes mitochondrial deenergization, alterations of H^+^ and Na^+^ homeostasis, and finally swelling of the sinusoidal endothelial cells (SEC), and the KC (12). Activation of KC with production of reactive oxygen species (ROS), upregulation of the inducible nitric oxide synthase (iNOS) in hepatocytes, and upregulation of proinflammatory cytokines, chemokines, and adhesion molecules resulting in neutrophil-mediated injury, are all major contributing events to the inflammation-associated damage (13). Below are the hepatic IRI mechanisms and mediators.

#### 3.1.1. ATP Depletion

Failure of aerobic ATP formation by oxidative phosphorylation is the fundamental stress of anoxic and ischemic injury. The importance of ATP depletion, in the events leading to necrotic cell death, is demonstrated by the ability of glycolytic substrates to rescue hepatocytes and sinusoidal endothelial cells from lethal cell injury (14). But glucose does not protect hepatocytes against anoxic injury since hepatocytes lack hexokinase. Endogenous glycogen is also an excellent substrate for anaerobic glycolysis (15). Fructose also prevents hepatocellular killing by several toxic chemicals, which implies that mitochondria are important targets of toxic cell killing (16). In addition, during anoxia, mitochondrial respiration, and hence oxidative phosphorylation become fully inhibited.

#### 3.1.2. ROS Creation, Kupffer Cells, and Chemokine Secretion

Reoxygenation of the hypoxic liver promotes the formation of ROS, including hydrogen peroxide (H_2_O_2_) and superoxide (O^2−^). The source of ROS in hepatic IRI has long been controversial. Regarding the mechanisms responsible for ROS production, experiments with xanthine oxidase (XOD)/xanthine dehydrogenase (XDH) inhibitors, such as allopurinol, suggest that the XOD/XDH system is the main ROS generator in hepatocytes (10). However, other results obtained in experimental models of the isolated perfused liver have suggested that the mitochondria, through the respiratory chain, could be the main source of ROS (17). Oxidative stress can damage cells through multiple mechanisms, including lipid peroxidation, DNA oxidation, and enzyme denaturation. It is also known that ROS do not cause cytotoxicity directly, but act as signaling molecules that upregulate nuclear transcription factors like Nuclear Factor kB (NF-kB) and subsequently release Tumor Necrosis Factor-a (TNF-α) and interleukin 1 (IL-1) (18). Additionally, ROS can cause damage from oxidant stress, which occurs during the early phase of injury, and activate inflammatory pathways that lead to neutrophil accumulation in the liver in the later phase. KCs are the resident macrophages of the liver and are critical in the earliest stages of IRI (19). They are responsible for the production of cytokines and chemokines which play a key role in the pathogenesis of IRI locally and systemically. However, the elimination of KC did not modify the deleterious effects of IRI and the activation of neutrophils is not essential for reoxygenation injury (20). TNF-a and IL-1 are the earliest cytokines to be increased in IRI, with increased levels occurring within minutes of reperfusion of the liver (21). Parenchymal cells of the liver may regulate the release of these cytokines during ischemic stress. Hepatocyte expression of interleukin-12 (IL-12) has been also shown to be a key element in the production of TNFa and IL-1 at reperfusion (22). The release of these molecules, which occurs from multiple cell types (KC, endothelial cells, hepatic parenchymal cells) finally results in a proinflammatory state caused by the resultant infiltration of immune cells in the injured organ (23). TNF-α is reported to have different effects in the IRI. It enhances oxidative stress-induced injury and induces apoptosis in hepatocytes, provided that protein synthesis or NF-ƙB-mediated gene expression is suppressed (24). At the same time, studies have linked disruption of TNF-α release to decreased hepatic injury and increased liver regeneration (25). Other mediators taking part in the injury process are interleukin 6 (IL-6) and glutathione levels. IL-6 release is delayed, and IL-6 treatment protects against warm IRI in rats (26). Decreased glutathione levels and elevated oxidized glutathione levels are used to demonstrate oxidative stress injury (27).

Additionally, IL-1 and TNF-α recruit and activate Cluster of Differentiation 4 (CD4^+^)T-lymphocytes, which produce granulocyte-macrophage colony-stimulating factor (GM-CSF), tumor necrosis factor β and γ (TNF-β and TNF-γ). These cytokines amplify KC activation and TNF-α and IL-1 secretion and promote neutrophil recruitment and adherence into the liver sinusoids, thus creating somewhat of a circular pattern in the activation process (11).

#### 3.1.3. Neutrophil Accumulation

Although chemokines serve to attract neutrophils to ischemic areas of injury via a chemokine gradient within the liver tissue, adhesion molecules such as intercellular adhesion molecule 1 (ICAM-1) with integrins such as CD11b/CD18 (also called Macrophage-1 antigen or MAC-1) are required for neutrophils to transmigrate from the bloodstream to the sites of injury (28, 29). Activation of neutrophils has been also implicated in the hepatic microvascular dysfunction and parenchymal damage associated with IRI (30). More specifically, the extensive vascular injury during reperfusion eliminates, in part, the sinusoidal endothelial cell barrier and the neutrophil has direct access to hepatocytes (31). When neutrophils infiltrate the liver, they worsen hepatic hypoperfusion, exacerbate the effects of Endothelin-1, and may release proteases through granule exocytosis (cathepsin G, granulocytes elastase) which are toxic to hepatocytes (31). In general, they perpetuate and amplify the injury by releasing many of the same mediators as KC, but often in much larger quantities.

#### 3.1.4. The Role of Nitric Oxide in IRI

The involvement of nitric oxide (NO) in IRI cannot be easily distinguished between beneficial or harmful. Some authors have found that NO exerts a beneficial effect on IRI in different organs, tissues and cells; whereas, other studies report no effect or even a deleterious action of NO (32). NO is formed by the enzyme iNOS from the precursor aminoacid arginine in the bloodstream. The endothelial form of NOS (eNOS) is constitutively expressed in endothelial cells of the liver and the iNOS form is induced during IRI and regulated by many of the same cytokines (TNFa and IL-1 among others) which are involved in IRI (33, 34). The production of NO during IRI seems to disturb the microcirculation of the liver and leads to mitochondrial dysfunction. Moreover, NO may promote apoptosis by inducing cytochrome c (Cyt-C) release and caspase activation, while it has been also shown to upregulate the antiapoptotic protein B-cell lymphoma 2 (Bcl-2) (35, 36).

#### 3.1.5. Apoptosis and Necrosis

During reperfusion, TNF-a and other mediators activate many of the proteins involved in apoptosis, such as the proteases caspase-3 and caspase-8, along with mitochondria Cyt-C release to the cytoplasm (37). This sequence of events leads to DNA destruction and apoptosis (38). The activation of caspases has also been used to demonstrate apoptosis in rat SECs following cold IRI (39). On the other hand, newer studies oppose the view that most cells undergo apoptosis in response to either warm or cold IRI, believing that necrosis is the principle form of cell death ( 12). The events that occur during the anoxia and the different phases of the reperfusion injury are being summarized in Table 1. 

**Table 1.** Key Events in the Pathophysiology of Hepatic IRI

Features
Decrease in oxidative phosphorylation results in ATP depletion
Derangement in Na^+^, Ca^+2^ and H^+^ homeostasis and mitochondrial deenergization
ROS ^a^ formation
Upregulation of NFkB factor and TNF-a-IL-1 release
Cytokines and Chemokines production by Kupffer cells
Activation of CD4^+ ^T helper cells
Neutrophil infiltration
NO ^a^ production
Hepatic cells apoptosis

^a^ Abbreviations: NO, nitric oxide; ROS, reactive oxygen species

### 3.2. Contemporary Approaches to Prevent Hepatic IRI

The predominant methods used to prevent the phenomenon of hepatic IRI are Ischemic Preconditioning (IP), in situ cooling of the liver and various pharmacological interventions. Furthermore, variations of existing liver clamping techniques, designed to reduce the IRI, have been extensively studied and are being routinely implemented during hepatic resections.

#### 3.2.1. Surgical Methods Used to Reduce Hepatic IRI

During liver resection, it is usually necessary to apply various selective or nonselective vascular occlusion techniques to reduce blood loss, as the latter is associated with adverse postoperative outcomes. These include the continuous or intermittent Pringle Maneuver (PM), the total hepatic vascular exclusion (THVE), and the hemihepatic or segmental occlusion of the portal vein or hepatic artery (40). Sadly, the aforementioned methods are accompanied by negative effects such as the hepatic IRI (6). Hence, surgeons need to achieve the right balance between reduced blood loss and attenuation of IRI.

The PM is the simplest and the most widely used and time-honored method of liver vascular clamping. It involves the simultaneous clamping of the hepatic artery and portal vein. By using intermittent, instead of continuous PM, hepatic IRI can be reduced, as intermittent clamping appears to be better tolerated (41). The optimal ischemic intervals are still debated and numerous studies try to define the optimal PM cycle. All in all, ischemic intervals of 30 minutes can be safely used followed by 5 minutes of reperfusion (40). According to der Broek et al. there were no significant differences between ischemic intervals of 15 minutes and 30 minutes as far as hepatocellular injury, median blood loss, liver function and morbidity are concerned (42). THVE has the advantage of less blood loss during the operation, while enabling difficult vascular reconstructions or reimplantations. It does have several adverse effects such as hemodynamic intolerance, increased operative time, and longer postoperative hospital stay compared to the PM (43, 44). Nevertheless, the combination of in situ Hypothermic Perfusion (HP) of the remnant liver with the THVE is promising. HP, which is achieved with cytoprotective solutions in combination with local cooling of the organ’s surface, has been used to prolong ischemic tolerance (45). Experimental studies have demonstrated that the parenchymal hypothermia reduces oxidative stress and the inflammatory response seen in IRI, thus allowing more complex hepatectomies to take place (46, 47).

##### 3.2.1.1. Ischemic Preconditioning

Regarding the IP most evidence comes from animal experiments and clinical series showing contrasting views (48). IP is characterized by a short period of ischemia and reperfusion preceding a longer time of ischemia. Three studies, in 2000, 2003 and 2004 respectively, demonstrated that IP was associated with significant beneficial effects and reduction of hepatic IRI, both in patients with steatotic and with normal liver parenchyma (49-51). Furthermore, IP prior to the PM can improve liver macro-circulation because it increases arterial perfusion and prevents portal vein postischemic flow reduction (52). On the contrary, a series of recent studies suggest that there is no additional protective effect of IP in patients undergoing liver resection under continuous or intermittent vascular occlusion (PM, intermittent PM or THVE) (53, 54). The most recent one by Jeon et al. showed that postoperative liver function tests, the duration of the operation, and the hospital stay were not significantly different between THVE alone and THVE preceded by IP. The morbidity rates were 37.5% for total vascular exclusion alone (THVE), and 34.2 % for THVE and IP, respectively. Moreover, the application of IP is not recommended in older patients (55, 56). However, using IP prior to intermittent PM is associated with reduced blood loss and shorter transection time (53).

#### 3.2.2. Pharmacological Approaches

Hepatic IRI has a complex pathophysiologic background. A lot of cell types, inflammatory mediators and reactive oxygen mediators play an important role. As a result, a lot of pharmacological interventions are being tested to reduce the phenomenon of IRI. 

Methylprednisolone, trimetazidine, glucose and ulinastatin may have protective roles against IRI in liver resection (57). Still, they remain controversial and cannot be routinely used to reduce IRI during controlled liver resections due to lack of clinical trials.

##### 3.2.2.1. Prednisolone

Prednisolone a glucocorticoid steroid, acts as an anti-inflammatory agent, reducing inflammatory markers and apoptotic cell count in experimental liver IRI (58). It decreased hospital stay and blood transfusion requirements, and improved liver function and showed a trend favoring a decreased postoperative complication rate (58). According to a 2013 systematic review and meta-analysis of the effect of perioperative steroids on IRI, perioperative steroids have a favorable impact on postoperative outcomes after liver resection. Patients receiving intravenous glucocorticoids were 24% less likely to have postoperative morbidity compared with controls. In addition, steroids significantly reduced postoperative blood levels of bilirubin and of inflammatory markers such as interleukin 6 (IL-6) and C-reactive protein (CRP). There was no evidence of a difference in infectious and wound healing complications between the study groups (59).

##### 3.2.2.2. Other Promising Pharmacological Interventions

Glucose, when given in high-concentration intravenously during 24 hrs before the operation, has proved to be beneficial in alleviating liver IRI during hepatic vascular occlusion (60). According to this study, hepatic tissue ATP content in the experimental group was significantly higher at the end of both the hepatic vascular occlusion and the point of the one hour reperfusion. Furthermore, the liver function of the experimental group was significantly better than that of the control group the first and fifth day after the operation. As far as ulinastatin is concerned, although it lowered the transaminase and bilirubin levels, it did not affect the rates of liver decompensation, perioperative morbidity or length of hospital stay (57, 61). Based on these results, it seems that ulinastatin may offer a protective role in elective liver resections under vascular occlusion. Another promising pharmacological substance is the Urinary Trypsin Inhibitor (UTI). UTI acts by reducing NF-κB activation and thus attenuates hepatic IRI. Wu YJ et al. demonstrated the potential effects of UTI administration before and after the liver ischemia (62). When UTI was administered preoperatively, lower Alanine transaminase and Aspartate transaminase levels were observed, as well as reduced NF-ƙB activation. The pathological hepatocellular damage was also improved, as neutrophil aggregation and infiltration, which lead to hepatic IRI, were inhibited (62). Last but not least, trimetazidine, an antianginal drug, may have a role in protecting the liver during resection under vascular occlusion. It has been shown to decrease liver I/R injury in experimental models by increasing ATP production and reducing oxygen consumption (58). Settaf et al. suggested that trimetazidine attenuates ischaemia-reperfusion injury during liver surgery (63). They demonstrated that the daily administration of trimetazidine five days prior to liver surgery, reduced cytolysis and increased liver ATP content. In addition, postoperative Aspartate transaminase (AST) and Alanine transaminase (ALT) levels and hospital stay were significantly lower in the trimetazidine group compared to the placebo group (63).

### 3.3. Potential Future Therapeutic Approaches to Prevent Hepatic IRI

The fact that hepatic cell damage, caused by IRI, can lead to severe complications, during or after liver transplantation or liver resections for hepatic trauma management, highlights the great need for research into this field, to gain more experience regarding the prevention of this undesirable effect.

The mechanisms responsible for hepatic IRI have been studied thoroughly and many attempts have been made to discover possible ways of successfully helping patients recover from major liver surgery. However, the fact that scientific research relies on the use of cell cultures and animal models, sets limitations on improving our knowledge on this subject, as most human tissues are not routinely accessible for research purposes. Despite the aforementioned difficulties, several methods about protecting the liver from IRI have been developed and tested in animal experimental models and significant efforts have been made to apply the results of these studies into clinical practice (1).

#### 3.3.1. Antioxidants

The possibility of ROS and oxidative stress being responsible for the cell damage occurring during IRI, has led to a large volume of recent research around antioxidant therapy. While the results in animal models are promising, there is a need for similar successes in human trials, so that these therapies can proceed to the clinical level.

#### 3.3.2. Endogenous Genes and Gene Products

Several gene products have proven to be protective when administered in the case of IRI. Generally, they tend to exert their effect mainly through the ROS (64). In this review, the use of glutathione and superoxide dismutase (SOD) would be discussed.

The tripeptide glutathione (GSH) is an antioxidant present in high concentrations in hepatocytes. Its levels are regulated by the Nuclear factor (erythroid-derived 2)-like 2 (Nrf 2) dependent gene glutaminate-cysteine ligase. It has been proven that intravenous administration of glutathione can effectively protect liver cells against reperfusion injury by detoxifying ROS (65). Studies have also demonstrated that treatment with the cysteine derivative, N-acetylcysteine (NAC), before or during IRI can maintain GSH levels and limit ROS. Furthermore, the fact that glutathione’s half life is short in humans has led to the thought that gene transfer would be able to increase intracellular glutathione levels without the need of intravenous administration in the future (64).

Another antioxidant enzyme that has been studied for its protective role against IRI, is SOD. Because of SOD’s poor bioavailability, the protection it offered, when administered intravenously, was not significant. On the other hand, when conjugated to carbohydrate factors, it proved to be much more effective. Gene transfer is an attractive option in this case too, but still at an early research stage (66).

#### 3.3.3. Mitochondrial Permeability Transition (MPT) inhibition:

The MPT plays an important role during hepatic damage by IRI, as it escalates the oxidative stress. Therefore, inhibitors of the MPT have proven to effectively protect hepatic cells. One such inhibitor is Edaravone, which is considered to reduce hepatic injury in the early stage of IRI, as shown in a porcine hepatectomy model (67).

#### 3.3.4. Pharmacological Preconditioning

As mentioned before, preconditioning is a procedure which involves exposing the liver to a brief period of ischemia and then reperfusion, before the actual period of hepatic ischemia. An alternative to the surgical preconditioning has been the effort to achieve pharmacologically preconditioning. Studies have shown that activating the Adenosine 2A (A2A) receptor using an A2A agonist, before the IRI, can protect against cell damage, as it mimics the physiological stimulation of the receptor that happens during IRI (68).

#### 3.3.5. Nitric Oxide

A substantial body of literature testifies to the fact that NO is an important endogenous molecule, involved in IRI. First of all, NO eliminates the decreased ATP levels related to liver (69) IRI. It also prevents the elevation of cytokine levels, such as TNF-a, interleukin β (IL-β) and IL-12, thereby decreasing the inflammatory process during which liver cell death occurs (70). As mentioned before, glutathione is associated with the protection of hepatic tissue during IRI, while NO mitigates oxidative damage by preventing reduction of this antioxidant (71). Last but not least, studies have shown that NO plays an important role in preserving the blood flow to the liver microcirculation during reperfusion, following a period of liver ischemia (69, 71). All these promising results have led to the hypothesis that exogenous NO could protect the liver. However, NO is a very unstable free radical and that is why its delivery needs another molecule to which NO would be attached to reach the liver. Some of the potential NO donors that could be used are S-nitrosothiols, diazeniumdiolates and liver-selective NO donors (72-76). All these molecules have been tested in numerous studies, with the S-nitrosothiols being the most studied of all.

#### 3.3.6. Nuclear Factor-kB (NF-ƙB)

NF-kB is an inducible nuclear transcription factor which regulates the expression of many genes and is also activated by a number of extracellular agents. NF-kB plays diverse roles in the acute injury response in both warm and cold hepatic ischemia. Activation of NF-kB in KC promotes inflammation through cytokine expression while activation in hepatocytes may be cell protective (77). With the multidimensional functions of NF-kB in reperfusion- injury, it should not come as a surprise that it has been implicated in numerous studies considering potential immunomodulatory therapeutic strategies. One of these studies has tried to determine whether the receptor activator of NF-kB (Receptor Activator of Nuclear Factor κB-RANK) and its ligand (RANKL) are important in the hepatic response to IRI, as it is well known that their interaction promotes NF-kB activation (78). The study proved that treatment with RANKL, before ischemia or at reperfusion, increased hepatocyte NF-kB activation, leading to a significant reduction in liver cell damage.

#### 3.3.7. Nilotinib

Even though antioxidants are the most studied solutions for preventing or reducing hepatic cell damage by IRI, there are other potential therapeutics. One of them is Nilotinib (receptor tyrosine kinase inhibitor), and its protective role against liver cell damage. One recent study has shown that Nilotinib lowers both liver Jun N-terminal kinases (JNK) activation and neural progenitor cells (NPC) p 38 mitogen-activated protein kinase (MAPK) activation in mice, and may be useful in ameliorating liver IRI in humans (79). All of the aforementioned methods are summarized in Table 2. 

**Table 2.** Current and Potential Options to Prevent Hepatic IRI

Current Surgical Options
Intermittent pringle maneuver
Hypothermic perfusion
Ischemic preconditioning
Current Pharmacological Options
Prednisolone
Glucose
Ulinastatin
Urinary trypsin inhibitor
Trimetazidine
Future Pharmacological Options
Glutatheione
Superoxide dismutases
Inhibitors of mitochondrial permeability transition
NO ^a^
NF-ƙB ^a^
Nilotinib

^a^ Abbreviations: NO, nitric oxide; NF-kB, nuclear factor kB

## 4. Conclusions

Hepatic IRI is an intriguing subject which still surprises with the countless mediators and interactions between them. It has an adverse effect on morbidity and mortality rates following liver resection and consequently should be seriously considered when dealing with hepatic trauma. The intermittent PM and the IP, along with the perioperative use of steroids and the antioxidant therapies, are some of the current options in our effort to reduce IRI. However, there has been extensive research in the field of noninvasive liver protection from IRI, mainly in animal models. Hopefully, this research would prove effective at introducing new methods of protection against IRI in the clinical practice.
